# Demographic recruitment bias of adults in United States randomized clinical trials by disease categories between 2008 to 2019: a systematic review and meta-analysis

**DOI:** 10.1038/s41598-022-23664-1

**Published:** 2023-01-02

**Authors:** Ilana Buffenstein, Bree Kaneakua, Emily Taylor, Masako Matsunaga, So Yung Choi, Enrique Carrazana, Jason Viereck, Kore Kai Liow, Arash Ghaffari-Rafi

**Affiliations:** 1 John A. Burns School of Medicine, University of Hawaiʻi at Mānoa, 651 Ilalo Street, Honolulu, HI 96813 USA; 2 Department of Quantitative Health Sciences, Biostatistics Core Facility, John A. Burns School of Medicine, University of Hawaiʻi at Mānoa, 651 Ilalo Street, Honolulu, HI 96813 USA; 3 Innovation and Translation Lab, Comprehensive Epilepsy Center, Hawaiʻi Pacific Neuroscience, 2230 Liliha St #104, Honolulu, HI 96817 USA; 4 Department of Neurological Surgery, School of Medicine, University of California, Davis, 4301 X St., Sacramento, CA 95817 USA

**Keywords:** Medical research, Clinical trial design

## Abstract

To promote health equity within the United States (US), randomized clinical trials should strive for unbiased representation. Thus, there is impetus to identify demographic disparities overall and by disease category in US clinical trial recruitment, by trial phase, level of masking, and multi-center status, relative to national demographics. A systematic review and meta-analysis were conducted using MEDLINE, Embase, CENTRAL, and ClinicalTrials.gov, between 01/01/2008 to 12/30/2019. Clinical trials (N = 5,388) were identified based on the following inclusion criteria: study type, location, phase, and participant age. Each clinical trial was independently screened by two researchers. Data was pooled using a random-effects model. Median proportions for gender, race, and ethnicity of each trial were compared to the 2010 US Census proportions, matched by age. A second analysis was performed comparing gender, race, and ethnicity proportions by trial phase, multi-institutional status, quality, masking, and study start year. 2977 trials met inclusion criteria (participants, n = 607,181) for data extraction. 36% of trials reported ethnicity and 53% reported race. Three trials (0.10%) included transgender participants (n = 5). Compared with 2010 US Census data, females (48.3%, 95% CI 47.2–49.3, *p* < 0.0001), Hispanics (11.6%, 95% CI 10.8–12.4, *p* < 0.0001), American Indians and Alaskan Natives (AIAN, 0.19%, 95% CI 0.15–0.23, *p* < 0.0001), Asians (1.27%, 95% CI 1.13–1.42, *p* < 0.0001), Whites (77.6%, 95% CI 76.4–78.8, *p* < 0.0001), and multiracial participants (0.25%, 95% CI 0.21–0.31, *p* < 0.0001) were under-represented, while Native Hawaiians and Pacific Islanders (0.76%, 95% CI 0.71–0.82, *p* < 0.0001) and Blacks (17.0%, 95% CI 15.9–18.1, *p* < 0.0001) were over-represented. Inequitable representation was mirrored in analysis by phase, institutional status, quality assessment, and level of masking. Between 2008 to 2019 representation improved for only females and Hispanics. Analysis stratified by 44 disease categories (i.e., psychiatric, obstetric, neurological, etc.) exhibited significant yet varied disparities, with Asians, AIAN, and multiracial individuals the most under-represented. These results demonstrate disparities in US randomized clinical trial recruitment between 2008 to 2019, with the reporting of demographic data and representation of most minorities not having improved over time.

## Introduction

Clinical trials have historically lacked equitable representation of people identifying as women and members of racial or ethnic minority groups^1^. Recognizing the issue, the National Institutes of Health (NIH), World Health Organization (WHO), and the United States (US) Food and Drug Administration (FDA) have improved reporting and inclusion of minorities, aiming for medical research to better reflect the shifting US demographics^2–6^. Nevertheless, significant disparities in representation persist^1,7–9^.

Prior systematic reviews and meta-analyses have been performed analyzing gender, ethnicity, and racial demographics in clinical trials for niche diseases (e.g. glaucoma, acute coronary syndrome, rheumatoid arthritis, dementia, congestive heart failure, cardiovascular, oncology, dyslipidemia), as well as for trials sponsored by select pharmaceutical groups^1,10–20^. However, data remains sparse on the inclusion of gender, ethnic, and racial groups in trials overall in the US, as well as by study phase, size, institutional status, masking, and trends in representation over time. Furthermore, past systematic reviews have included multi-institutional studies with international locations, which can limit the ability to accurately reflect US demographics^10,15,21^.

In addition, despite policies that seek to address enrollment and recruitment in clinical trials, longitudinal data regarding inclusion of women and minorities in trials overall has not been assessed since the passage of these initiatives^1–6^. Using available ClinicalTrials.gov demographic data, our study assessed whether adult women and minorities were underrepresented in US phase 2 and 3 randomized clinical trials between 2008 and 2019, comparing demographic proportions overall and within disease categories (i.e., psychiatric disorders, obstetric/gynecologic, neurological, cardiovascular, etc.), by study phase, trial quality tier, institutional status, level of masking, and study start year.

## Methods

**Figure 1:**
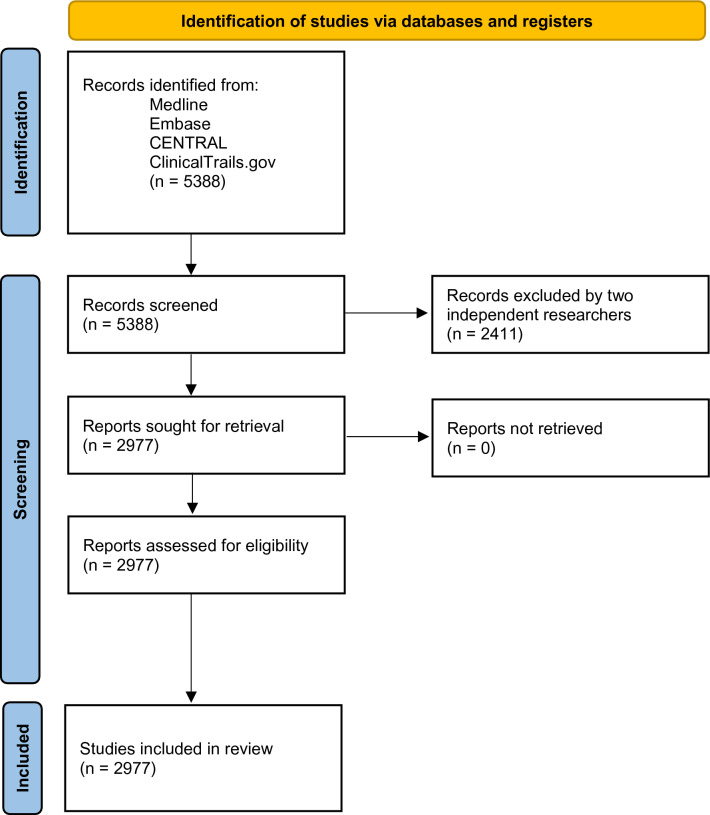
PRISMA flowchart.

Using the PRISMA (Preferred Reporting Items for Systematic Reviews and Meta-Analyses) guidelines and the Cochrane Handbook of Systematic Reviews of Interventions, we conducted a systematic review through MEDLINE, Embase, Cochrane Central Register of Controlled Trials (CENTRAL), and ClinicalTrials.gov. (Fig. 1).

### Data collection

Clinical trials were identified utilizing the US Clinical Trial Registry website (https://clinicaltrials.gov/). Clinical trials (N = 5388) were reviewed based on the following criteria: study type (interventional and randomized), minimum participant age (18 years old), study location (US, including Puerto Rico), phase (2 and/or 3), study status (completed or active/not recruiting new participants), start date (01/01/2008 to 12/30/2019), and results date (on or before 11/18/2020). Each clinical trial was screened independently by two reviewers. Multi-center studies including international locations were excluded. Discrepancies between reviewers for inclusion of specific trials were resolved through discussion and a third reviewer.

Data was then extracted from the clinical trials which met inclusion criteria (N = 2977). Extraction was performed independently by two reviewers and then compared for consensus. Data extracted from each trial included: start date, phase, level of masking, total number of participants, multi-center status (multi-center or single institution), number of participants of each gender (male, female, or transgender), ethnicity group (Hispanic or non-Hispanic), and racial group (American Indian and Alaskan Native [AIAN], Asian, Native Hawaiian and Pacific Islander [NHPI], Black and African American [Black], White, or more than one race [multiracial]), as well as whether race and ethnicity data were reported by the trial. Studies that were characterized as combined phases were categorized to the higher phase (i.e. trials having completed phases 1 and 2 were categorized as phase 2; trials having completed phases 2 and 3 were categorized as phase 3) ^21^.

A two-tier assessment was performed for each trial based on the following criteria (one point for each parameter): (1) multi-center; (2) ≥ 200 participants; (3) reports ethnicity; (4) reports race. Trials scoring 0, 1, or 2 points were assigned as tier 1. Trials scoring 3 or 4 points were assigned as tier 2. Tier assessment was based on Cochrane Library guidelines and the Hoy Risk of Bias Tool, but modified in that tier assigned was based on objective data available from ClinicalTrials.gov^22,23^. Discrepancies in individual assessments of tier were resolved through discussion and involvement of a third reviewer.

Trial participants labeled as *unknown, other, missing, or not reported* within the gender (individual participants, n = 190), race (n = 7437), and ethnicity (n = 2468) categories were excluded from each group’s total proportion. 0.10% of trials (N = 3) included gender minorities, therefore transgender trial participants (n = 5) were excluded from each trial’s total gender proportion for statistical analysis.

### Statistical analysis

For full dataset analysis, as well as disease strata, we compared trial median proportions for gender, race, and ethnicity to 2010 official US Census proportions matched by age (≥ 18 years old) using Wilcoxon rank sum analysis^24^. For analysis of gender, random effects modeling meta-analyses were performed for female representation overall and for female representation excluding disease categories with significant gender skew. A secondary analysis was performed comparing gender, race, and ethnicity proportions by trial phase, multi-institutional status, trial tier, masking, and study start year. Trial median proportions for gender, ethnicity, and race trended by study start year were compared to the reference year of 2008 using Wilcoxon rank sum analysis and random effects modeling for the meta-analysis. Funnel plots were developed to examine scatter patterns of trial proportions surrounding the summary proportion by number of trial participants for gender, ethnicity, and race. All analyses were conducted via the R Statistical Software (R Foundation for Statistical Computing, Vienna, Austria). The systematic review and meta-analysis were registered on PROSPERO, with the identifier of CRD42021238101.

## Results

### Gender

Three clinical trials (0.10%) from 2977 reported inclusion of transgender participants (total participants, n = 5). These trials examined treatment of major depressive disorder, anal neoplasms, and human immunodeficiency virus (HIV).

### Female representation

**Figure 2:**
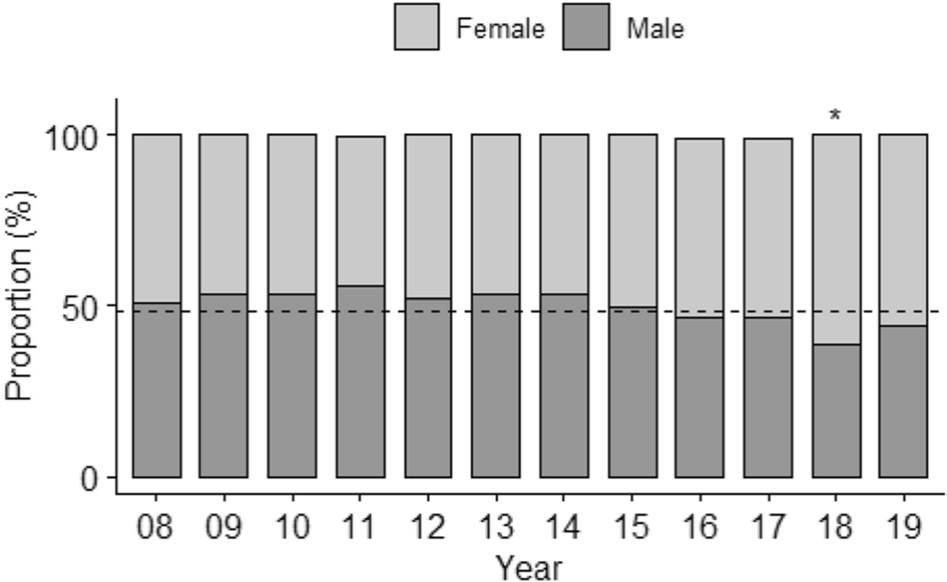
Female and Male Representation Trended Between 2008–2019. Median proportions of gender groups (%) by year (N = 2693). The dashed line is the male proportion from Census 2010. *P < 0.05 (Wilcoxon rank sum test comparing annual female proportion with the 2008 clinical trial proportion).

After excluding disease categories with significant gender recruitment skew (i.e., pregnancy, prostate cancer, etc.), females were found underrepresented (48.3%, 95% CI 47.2–49.3) overall when compared to the US Census proportion (51.5%, *p* < 0.0001). However, between 2008 and 2018 representation increased (*p* = 0.0005), with females being overrepresented in 2018 (64.0%, 95% CI 56.5–71.2; *p* = 0.0012) (Fig. 2).

When examining clinical trial phases, females were underrepresented in phase 2 (46.2%, 95% CI 44.9–47.5; *p* < 0.0001), yet accurately represented in phase 3 (52.3%, 95% CI 50.5–54.0; *p* = 0.41), with the proportion significantly increasing between phase 2 to 3 (*p* < 0.0001). Regardless of a trial’s institutional status, females were underrepresented in both single institution (47.2%, 95% CI 45.7–48.7; *p* < 0.0001) and multi-institutional (49.2%, 95% CI 47.8–50.6; *p* = 0.0017) trials, with representation similar between both groups (*p *= 0.065). Tier 2 trials exhibited appropriate representation (50.5%, 95% CI 48.5–52.5; *p* = 0.33), unlike tier 1 (47.2%, 95% CI 45.7–48.7; *p* < 0.0001). For masking status, females were underrepresented in trials with no (42.6%, 95% CI 40.2–45.0; *p* < 0.0001), single (46.6%, 95% CI 42.8–50.4; *p* = 0.012), and quadruple (48.7%, 95% CI 46.8–50.6; *p* = 0.0032) masking, while accurately represented in trials with double (51.0%, 95% CI 48.9–53.1; *p* = 0.63) and triple (50.0%, 95% CI 47.7–52.4; *p* = 0.22) masking. Relative to trials with no masking, female proportions were greater in trials with double (*p* < 0.0001), triple (*p* = 0.0001), and quadruple (*p* = 0.0001) masking (Table 1).

**Table 1:** Female proportion estimates overall, by phase, multi-institutional status, tier assessment, masking, and year. 284 studies from diseases related to prostate cancer (54 trials), breast cancer (60 trials), gynecologic cancer (21 trials), female genitourinary diseases (73 trials), male genitourinary diseases (36 trials), and pregnancy (40 trials) were excluded from this analysis (N = 2693).

	Frequency (%), studies	Total number of participants	Estimated proportion (95% CI)	*P*-value compared to reference category (*)	*P*-value compared to 2010 Census proportion (51.5%)	${I}^{2}$(%)
*Female*						
Overall	–	–	48.3% (47.2–49.3)	–	< 0.0001	98.3%
*Phase*						
II*	1807 (67.1%)	178,034	46.2% (44.9–47.5)	–	< 0.0001	98.2%
III	886 (32.9%)	380,015	52.3% (50.5–54.0)	< 0.0001	0.41	
*Institution status*						
Single*	1327 (49.3%)	185,144	47.2% (45.7–48.7)	–	< 0.0001	98.2%
Multi-institutional	1366 (50.7%)	372,905	49.2% (47.8–50.6)	0.065	0.0017	
*Tier assessment*						
1*	1997 (74.2%)	300,740	47.4% (46.2–48.7)	–	< 0.0001	98.3%
2	696 (25.8%)	257,309	50.5% (48.5–52.5)	0.011	0.33	
*Masking*						
No masking*	489 (18.2%)	83,289	42.6% (40.2–45.0)	–	< 0.0001	98.2%
Single	203 (7.54%)	27,182	46.6% (42.8–50.4)	0.079	0.012	
Double	661 (24.6%)	131,845	51.0% (48.9–53.1)	< 0.0001	0.63	
Triple	518 (19.2%)	116,862	50.0% (47.7–52.4)	0.0001	0.22	
Q uadruple	822 (30.5%)	198,871	48.7% (46.8–50.6)	0.0001	0.0032	
*Study start year*						
2008*	388 (14.4%)	88,016	49.6% (46.9–52.3)	–	0.17	98.2%
2009	357 (13.3%)	68,633	45.9% (43.1–48.7)	0.063	0.0001	
2010	304 (11.3%)	72,958	45.7% (42.3–49.0)	0.098	0.0007	
2011	257 (9.54%)	37,988	45.7% (42.3–49.0)	0.072	0.0006	
2012	278 (10.3%)	55,467	46.0% (42.8–49.2)	0.092	0.0008	
2013	266 (9.88%)	50,085	46.7% (43.4–50.0)	0.18	0.0045	
2014	257 (9.54%)	64,981	45.5% (42.2–48.9)	0.062	0.0004	
2015	235 (8.73%)	42,550	51.6% (48.1–55.2)	0.37	0.94	
2016	161 (5.98%)	41,402	53.1% (48.8–57.3)	0.18	0.47	
2017	126 (4.68%)	22,836	55.8% (51.0–60.5)	0.03	0.082	
2018	50 (1.86%)	9947	64.0% (56.5–71.2)	0.0005	0.0012	
2019	14 (0.52%)	3186	56.4% (41.9–70.3)	0.37	0.51	

### Ethnicity

Of the 2977 trials, 35.7% (N = 1062) reported ethnicity, with 0.4% of participants (n = 2468) having their ethnicity reported as *unknown*.

#### Hispanic

**Figure 3:**
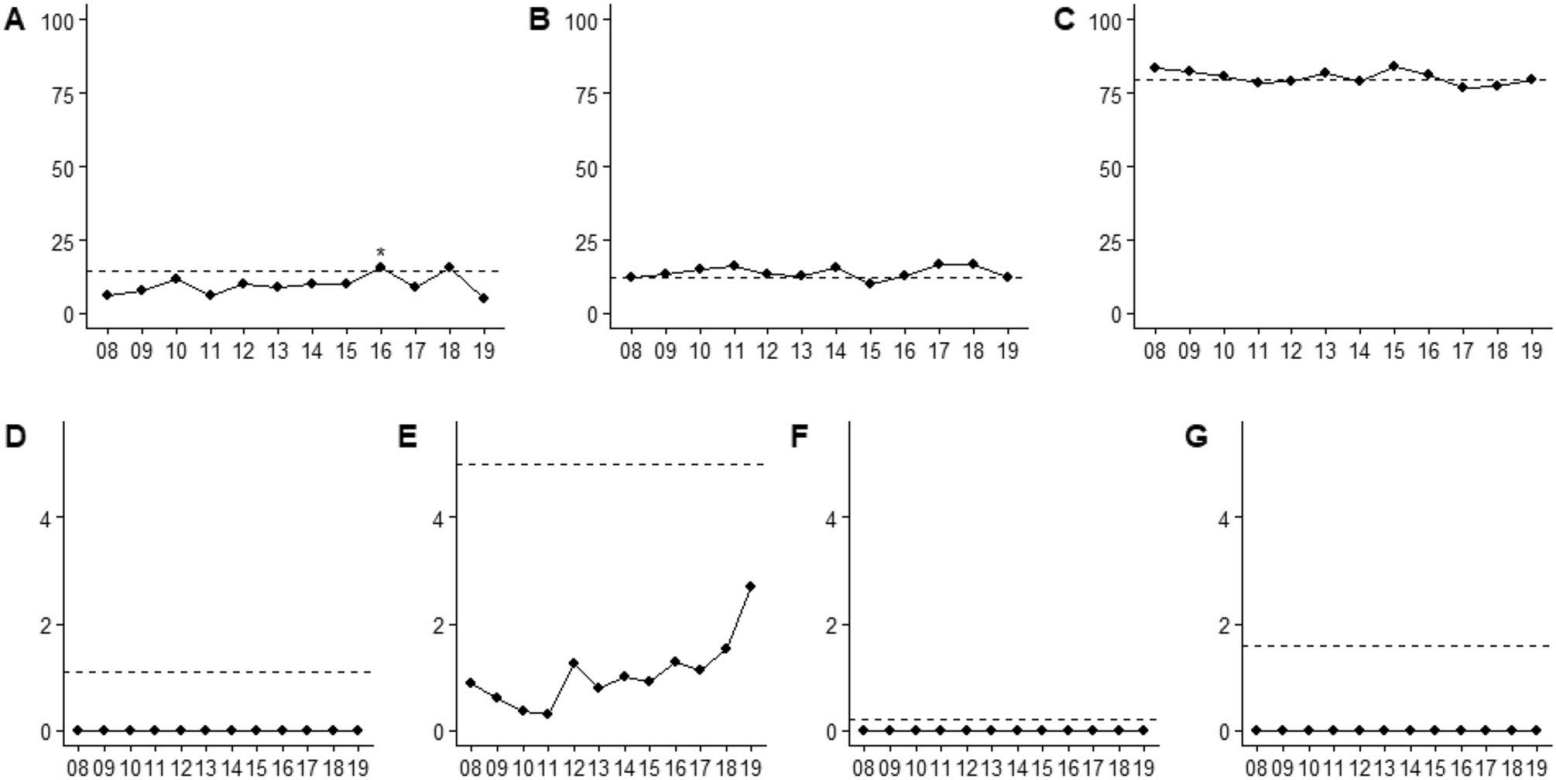
Ethnicity and Race Trended Between 2008–2019. Median proportions (%) of Hispanics (N = 1062) and race categories (N = 1589) by year, with the x-axis representing the year (2008–2019) and the y-axis the ethnicity/race proportion (%). (**A**): Hispanics, *P < 0.05 (Wilcoxon rank sum test comparing annual proportion relative to 2008 clinical trial proportion); (**B**): Blacks; (**C**): Whites; (**D**): American Indians and Alaskan Natives; (**E**): Asian; (**F**): Native Hawaiians and Pacific Islanders; (**G**): Multiracial individuals. The dashed line represents the designed demographic proportion from Census 2010.

In trials reporting ethnicity, Hispanics were underrepresented (11.6%, 95% CI 10.8–12.4; *p *< 0.0001) overall, relative to the Census proportion (14.2%) (Table 2). Yet between 2008 and 2016 representation significantly increased (*p* < 0.0001), where Hispanics were over-represented in trials started in 2016 (18.7%, 95% CI, 15.8–21.7; *p* = 0.0003) (Fig. 3A).

**Table 2:** Hispanic proportion estimates overall, by phase, multi-institutional status, tier assessment, masking, and year.

	Frequency (%), studies	Total number of participants	Estimated proportion (95% CI)	*P*-value compared to reference category (*)	*P*-value compared to 2010 Census proportion (14.2%)	${I}^{2}$(%)
*Hispanic*						
Overall	–	–	11.6% (10.8–12.4)	–	< 0.0001	97.3%
*Phase*						
II*	704 (66.3%)	74,942	10.6% (9.63–11.6)	–	< .0001	97.3%
III	358 (33.7%)	182,845	13.5% (12.0–15.0)	0.0012	0.97	
*Institution status*						
Single*	414 (39.0%)	85,147	10.8% (9.51–12.2)	–	0.0001	97.3%
Multi-institutional	648 (61.0%)	172,640	12.1% (11.0–13.1)	0.14	0.0093	
*Tier assessment*						
1*	371 (34.9%)	19,709	10.3% (8.96–11.7)	–	< .0001	97.3%
2	691 (65.1%)	238,078	12.2% (11.2–13.3)	0.030	0.017	
*Masking*						
No masking*	218 (20.5%)	40,976	10.3% (8.61–12.1)	–	0.0008	97.2%
Single	73 (6.87%)	18,956	11.1% (8.17–14.3)	0.67	0.14	
Double	259 (24.4%)	57,691	11.0% (9.42–12.7)	0.57	0.0039	
Triple	198 (18.6%)	61,581	13.6% (11.6–15.7)	0.016	0.93	
Quadruple	314 (29.6%)	78,583	11.9% (10.4–13.4)	0.19	0.041	
*Study start year*						
2008*	76 (7.16%)	22,365	9.14% (6.57–12.1)	–	0.0041	97.0%
2009	87 (8.19%)	21,394	8.75% (06.37–11.4)	0.84	0.0008	
2010	85 (8.00%)	41,081	11.8% (9.08–14.8)	0.19	0.27	
2011	99 (9.32%)	12,522	9.52% (7.19–12.1)	0.84	0.0032	
2012	105 (9.89%)	25,760	10.3% (7.92–12.9)	0.55	0.015	
2013	125 (11.8%)	27,190	11.1% (8.81–13.5)	0.30	0.049	
2014	121 (11.4%)	19,701	10.3% (8.13–12.7)	0.52	0.010	
2015	121 (11.4%)	24,943	12.2% (9.81–14.7)	0.11	0.30	
2016	112 (10.6%)	35,195	18.7% (15.8–21.7)	< .0001	0.0003	
2017	92 (8.66%)	16,547	13.5% (10.7–16.6)	0.034	0.99	
2018	27 (2.54%)	8175	14.1% (9.10–19.9)	0.096	0.83	
2019	12 (1.13%)	2914	10.2% (3.89–18.7)	0.80	0.41

While Hispanics were underrepresented in phase 2 trials (10.6%, 95% CI 9.63–11.6; *p* < 0.0001) the proportion significantly increased between phase 2 and 3 (*p* = 0.0012), with appropriate representation in phase 3 (13.5%, 95% CI 12.0–15.0; *p* = 0.972). For institutional status, there was underrepresentation in both single (10.8%, 95% CI 9.51–12.2; *p* < 0.0001) and multi-institutional (12.1%, 95% CI 11.0–13.1; *p* = 0.0093) trials. Similarly. regardless of trial tier, Hispanics were underrepresented in both tier 1 (10.3%, 95% CI 8.96–11.7; *p* < 0.0001) and tier 2 (12.2%, 95% CI 11.2–13.3; *p* = 0.017), yet representation increased between the first and second tiers (*p* = 0.03). For masking, Hispanics were underrepresented in trials with no (10.3%, 95% CI 8.61–12.1; *p* = 0.0008), double (11.0%, 95% CI 9.42–12.7; *p* = 0.0039), and quadruple masking (11.9%, 95% CI 10.4–13.4; *p* = 0.041), while accurately represented in single (11.1%, 95% CI 8.17–14.3; *p* = 0.14) and triple masking (13.6%, 95% CI 11.6–15.7; *p* = 0.93).

### Race

From the 2977 clinical trials, 53% reported race (N = 1589), with 1% of participants having their race reported as *unknown* (n = 7437). Between 2008 and 2019, the proportions of all racial strata did not significantly change (Table 2–7).

#### American Indian and Alaska native

AIAN were underrepresented (0.19%, 95% CI 0.15–0.23; *p* < 0.0001) in trials overall, relative to the Census proportion (1.10%) (Table 3). In both phase 2 (0.13%, 95% CI 0.10–0.18; *p* < 0.0001) and 3 (0.30%, 95% CI 0.23–0.38; *p* < 0.0001), AIAN were underrepresented, yet representation increased between phase 2 to 3 (*p* < 0.0001). While AIAN were underrepresented regardless of institutional status of the trial, there was a significant increase (*p* < 0.0001) in representation between single (0.09%, 95% CI 0.10–0.18) and multi-institutional (0.26%, 95% CI 0.21–0.31) studies. For trial tier, AIAN were underrepresented in both tier 1 (0.09%, 95% CI 0.05–0.13; *p* < 0.0001) and 2 trials (0.29%, 95% CI 0.23–0.35; *p* < 0.0001), however representation was significantly greater in tier 2 relative to 1 (*p* < 0.0001). Regardless of masking status, AIAN remained underrepresented (*p* < 0.0001), with representation similar amongst all degrees of masking.

**Table 3:** American Indian and Alaska Native proportion estimates overall, by phase, multi-institutional status, tier assessment, masking, and year.

	Frequency (%), studies	Total number of participants	Estimated proportion (95% CI)	*P*-value compared to reference category (*)	*P*-value compared to 2010 Census proportion (1.10%)	${I}^{2}$(%)
*American Indian and Alaska Native*						
Overall	–	–	0.19% (0.15–0.23)	–	< 0.0001	78.5%
*Phase*						
II*	1094 (68.9%)	103,813	0.13% (0.10–0.18)	–	< .0001	78.2%
III	495 (31.2%)	220,155	0.30% (0.23–0.38)	< .0001	< .0001	
*Institution status*						
Single*	682 (42.9%)	102,520	0.09% (0.05–0.14)	–	< .0001	77.9%
Multi-institutional	907 (57.1%)	221,448	0.26% (0.21–0.31)	< .0001	< .0001	
*Tier assessment*						
1*	824 (51.9%)	57,943	0.09% (0.05–0.13)	–	< .0001	78.0%
2	765 (48.1%)	266,025	0.29% (0.23–0.35)	< .0001	< .0001	
*Masking*						
No masking*	341 (21.5%)	55,000	0.20% (0.12–0.29)	–	< .0001	78.0%
Single	107 (6.73%)	22,081	0.13% (0.03–0.27)	0.357	< .0001	
Double	389 (24.5%)	79,017	0.17% (0.11–0.25)	0.645	< .0001	
Triple	287 (18.1%)	72,527	0.22% (0.13–0.32)	0.746	< .0001	
Quadruple	465 (29.3%)	95,343	0.20% (0.14–0.28)	0.981	< .0001	
*Study start year*						
2008*	121 (7.61%)	35,685	0.13% (0.04–0.26)	–	< .0001	77.5%
2009	156 (9.82%)	30,239	0.27% (0.15–0.43)	0.135	< .0001	
2010	134 (8.43%)	45,381	0.22% (0.10–0.38)	0.337	< .0001	
2011	145 (9.13%)	17,765	0.14% (0.05–0.27)	0.891	< .0001	
2012	159 (10.0%)	29,668	0.24% (0.12–0.39)	0.219	< .0001	
2013	189 (11.9%)	32,347	0.11% (0.04–0.21)	0.639	< .0001	
2014	169 (10.6%)	25,349	0.17% (0.08–0.30)	0.616	< .0001	
2015	177 (11.1%)	29,085	0.31% (0.18–0.48)	0.0525	< .0001	
2016	150 (9.44%)	41,432	0.19% (0.09–0.32)	0.490	< .0001	
2017	128 (8.06%)	23,619	0.14% (0.05–0.29)	0.868	< .0001	
2018	47 (2.96%)	10,243	0.20% (0.04–0.49)	0.545	< .0001	
2019	14 (0.88%)	3155	0.20% (0.00–0.87)	0.737	0.0156	

#### Asian

Relative to the Census (5.01%), Asians were underrepresented (1.27%, 95% CI 1.13–1.42; *p* < 0.0001) in clinical trials overall, regardless of trial phase, institutional status, tier, and masking classification (Table 4). While representation was similar between phase 2 and 3 (*p* = 0.98), as well as single and multi-institutional status (*p* = 0.31), trials classified as tier 2 exhibited greater representation than tier 1 (*p* = 0.0045), and in trials with triple masking representation was greater than those with no masking (*p* = 0.045).

**Table 4:** Asian proportion estimates overall, by phase, multi-institutional status, tier assessment, masking, and year.

	Frequency (%), studies	Total number of participants	Estimated proportion (95% CI)	*P*-value compared to reference category (*)	*P*-value compared to 2010 Census proportion (91.3%)	${I}^{2}$(%)
*Asian*						
Overall	–	–	1.27% (1.13–1.42)	–	< 0.0001	91.3%
*Phase*						
II*	1094 (68.9%)	103,813	1.27% (1.10–1.45)	–	< .0001	91.3%
III	495 (31.2%)	220,155	1.27% (1.04–1.52)	0.979	< .0001	
*Institution status*						
Single*	682 (42.9%)	102,520	1.18% (0.96–1.41)	–	< .0001	91.2%
Multi-institutional	907 (57.1%)	221,448	1.33% (1.15–1.52)	0.308	< .0001	
*Tier assessment*						
1*	824 (51.9%)	57,943	1.05% (0.87–1.26)	–	< .0001	91.2%
2	765 (48.1%)	266,025	1.47% (1.27–1.68)	0.0045	< .0001	
*Masking*						
No masking*	341 (21.5%)	55,000	1.16% (0.88–1.47)	–	< .0001	91.0%
Single	107 (6.73%)	22,081	0.78% (0.40–1.28)	0.218	< .0001	
Double	389 (24.5%)	79,017	1.50% (1.20–1.83)	0.119	< .0001	
Triple	287 (18.1%)	72,527	1.65% (1.29–2.05)	0.0447	< .0001	
Quadruple	465 (29.3%)	95,343	1.08% (0.85–1.34)	0.709	< .0001	
*Study start year*						
2008*	121 (7.61%)	35,685	1.19% (0.75–1.73)	–	< .0001	90.8%
2009	156 (9.82%)	30,239	0.95% (0.60–1.38)	0.438	< .0001	
2010	134 (8.43%)	45,381	0.81% (0.47–1.25)	0.226	< .0001	
2011	145 (9.13%)	17,765	0.88% (0.53–1.32)	0.332	< .0001	
2012	159 (10.0%)	29,668	1.37% (0.94–1.88)	0.616	< .0001	
2013	189 (11.9%)	32,347	1.24% (0.86–1.69)	0.534	< .0001	
2014	169 (10.6%)	25,349	1.32% (0.91–1.80)	0.715	< .0001	
2015	177 (11.1%)	29,085	1.45% (1.02–1.95)	0.460	< .0001	
2016	150 (9.44%)	41,432	1.65% (1.17–2.20)	0.216	< .0001	
2017	128 (8.06%)	23,619	1.96% (1.38–2.64)	0.0585	< .0001	
2018	47 (2.96%)	10,243	1.57% (0.78–2.62)	0.465	< .0001	
2019	14 (0.88%)	3155	1.55% (0.30–3.77)	0.683	0.0051	

#### Native Hawaiian and Pacific Islander

NHPI were overrepresented (0.76%, 95% CI 0.71–0.82; *p* < 0.0001) in trials overall (Census: 0.20%), regardless of phase, institutional status, tier, and masking classification (Table 5). The NHPI proportion was significantly lower in phase 3 versus 2 (*p* < 0.0001), multi-institutional versus single (*p* < 0.0001), and in tier 2 versus 1 (*p* < 0.0001). NHPI proportion was significantly greater in trials with single masking, relative to none (*p* = 0.017).

**Table 5:** Native Hawaiian and Pacific Islander proportion estimates overall, by phase, multi-institutional status, tier assessment, masking, and year.

	Frequency (%), studies	Total number of participants	Estimated proportion (95% CI)	*P*-value compared to reference category (*)	*P*-value compared to 2010 Census proportion (0.20%)	${I}^{2}$(%)
*Native Hawaiian and Pacific Islander*						
Overall	–	–	0.76% (0.71–0.82)	–	< 0.0001	31.3%
*Phase*						
II*	1094 (68.9%)	103,813	1.03% (0.95–1.12)	–	< .0001	30.1%
III	495 (31.2%)	220,155	0.46% (0.41–0.52)	< .0001	< .0001	
*Institution status*						
Single*	682 (42.9%)	102,520	1.15% (1.03–1.29)	–	< .0001	30.1%
Multi-institutional	907 (57.1%)	221,448	0.60% (0.55–0.65)	< .0001	< .0001	
*Tier assessment*						
1*	824 (51.9%)	57,943	1.21% (1.09–1.33)	–	< .0001	30.9%
2	765 (48.1%)	266,025	0.53% (0.48–0.58)	< .0001	< .0001	
*Masking*						
No masking*	341 (21.5%)	55,000	0.75% (0.64–0.87)	–	< .0001	29.0%
Single	107 (6.73%)	22,081	1.08% (0.83–1.39)	0.0171	< .0001	
Double	389 (24.5%)	79,017	0.69% (0.60–0.79)	0.472	< .0001	
Triple	287 (18.1%)	72,527	0.84% (0.71–0.98)	0.317	< .0001	
Quadruple	465 (29.3%)	95,343	0.74% (0.66–0.84)	0.978	< .0001	
*Study start year*						
2008*	121 (7.61%)	35,685	0.66% (0.52–0.85)	–	< .0001	28.1%
2009	156 (9.82%)	30,239	0.59% (0.47–0.74)	0.473	< .0001	
2010	134 (8.43%)	45,381	0.80% (0.63–1.00)	0.292	< .0001	
2011	145 (9.13%)	17,765	0.78% (0.62–0.98)	0.359	< .0001	
2012	159 (10.0%)	29,668	0.84% (0.68–1.04)	0.151	< .0001	
2013	189 (11.9%)	32,347	0.75% (0.61–0.92)	0.898	< .0001	
2014	169 (10.6%)	25,349	0.94% (0.76–1.15)	0.0367	< .0001	
2015	177 (11.1%)	29,085	0.85% (0.70–1.05)	0.127	< .0001	
2016	150 (9.44%)	41,432	0.66% (0.54–0.81)	0.968	< .0001	
2017	128 (8.06%)	23,619	0.75% (0.59–0.95)	0.505	< .0001	
2018	47 (2.96%)	10,243	0.87% (0.59–1.30)	0.245	< .0001	
2019	14 (0.88%)	3155	0.88% (0.45–1.73)	0.439	0.0074	

#### Black

In relation to the Census proportion (12.3%), Blacks were overrepresented (17.0%, 95% CI 15.9–18.1; *p* < 0.0001) overall in clinical trials, regardless of phase, institutional status, trial tier, and masking classification (*p* < 0.0001) (Table 6). Single institutional trials had greater representation than multi-institutional (*p* = 0.0002). Meanwhile, compared with no masking, representation was significantly greater in trials with single (*p* = 0.0005), triple (*p* = 0.0065), and quadruple (*p* = 0.0077) masking.

**Table 6:** Black proportion estimates overall, by phase, multi-institutional status, tier assessment, masking, and year.

	Frequency (%), studies	Total number of participants	Estimated proportion (95% CI)	*P*-value compared to reference category (*)	*P*-value compared to 2010 Census proportion (12.3%)	${I}^{2}$(%)
*Black*						
Overall	–	–	17.0% (15.9–18.1)	–	< 0.0001	98.3%
*Phase*						
II*	1094 (68.9%)	103,813	16.5% (15.3–17.8)	–	< .0001	98.3%
III	495 (31.2%)	220,155	18.0% (16.1–19.9)	0.220	< .0001	
*Institution status*						
Single*	682 (42.9%)	102,520	19.4% (17.7–21.2)	–	< .0001	98.3%
Multi-institutional	907 (57.1%)	221,448	15.3% (14.0–16.7)	0.0002	< .0001	
*Tier assessment*						
1*	824 (51.9%)	57,943	17.9% (16.4–19.5)	–	< .0001	98.3%
2	765 (48.1%)	266,025	16.1% (14.6–17.6)	0.091	< .0001	
*Masking*						
No masking*	341 (21.5%)	55,000	13.9% (11.8–16.1)	–	< .0001	98.3%
Single	107 (6.73%)	22,081	22.4% (18.1–27.1)	0.0005	< .0001	
Double	389 (24.5%)	79,017	16.2% (14.1–18.4)	0.130	< .0001	
Triple	287 (18.1%)	72,527	18.5% (16.0–21.1)	0.0065	< .0001	
Quadruple	465 (29.3%)	95,343	17.9% (15.9–19.9)	0.0077	< .0001	
*Study start year*						
2008*	121 (7.61%)	35,685	13.7% (10.4–17.4)	–	0.200	98.2%
2009	156 (9.82%)	30,239	14.5% (11.5–17.8)	0.738	0.0500	
2010	134 (8.43%)	45,381	21.0% (17.2–25.0)	0.0070	< .0001	
2011	145 (9.13%)	17,765	22.5% (18.8–26.5)	0.0010	< .0001	
2012	159 (10.0%)	29,668	17.3% (14.0–20.7)	0.155	0.0003	
2013	189 (11.9%)	32,347	15.1% (12.3–18.2)	0.545	0.0107	
2014	169 (10.6%)	25,349	18.6% (15.4–22.1)	0.0495	< .0001	
2015	177 (11.1%)	29,085	14.5% (11.6–17.6)	0.749	0.0427	
2016	150 (9.44%)	41,432	14.5% (11.4–17.8)	0.753	0.0584	
2017	128 (8.06%)	23,619	19.1% (15.3–23.2)	0.0441	< .0001	
2018	47 (2.96%)	10,243	20.7% (14.5–27.7)	0.0584	0.0026	
2019	14 (0.88%)	3155	15.8% (6.31–28.2)	0.719	0.419	

#### White

Overall, in clinical trials Whites were underrepresented (77.6%, 95% CI 76.4–78.8; *p* < 0.0001) when compared to the Census proportion (79.8%), irrespective of trial phase or tier (Table 7). However, multi-institutional trials exhibited appropriate representation (80.0%, 95% CI 78.5–81.5; *p* = 0.52), unlike single-institutional trials where Whites were underrepresented (74.1%, 95% CI 74.1–76.1; *p* < 0.0001). In trials with single (71.8%, 95% CI 66.7–76.6; *p* = 0.0003), triple (74.0%, 95% CI 71.0–76.9; *p* < 0.0001), and quadruple masking (77.2%, 95% CI 75.0–79.4; *p* = 0.0031) Whites were also underrepresented, while in trials with no (81.6%, 95% CI 79.1–83.9; *p* = 0.396) and double masking (78.7%, 95% CI 76.3–81.0; *p *= 0.136) there was accurately representation.

**Table 7:** White proportion estimates overall, by phase, multi-institutional status, tier assessment, masking, and year.

	Frequency (%), studies	Total number of participants	Estimated proportion (95% CI)	*P*-value compared to reference category (*)	*P*-value compared to 2010 Census proportion (79.8%)	${I}^{2}$(%)
*White*						
Overall	–	–	77.6% (76.4–78.8)	–	< 0.0001	98.4%
*Phase*						
II*	1094 (68.9%)	103,813	78.0% (76.6–79.5)	–	0.0007	98.4%
III	495 (31.2%)	220,155	76.7% (74.5–78.8)	0.31	0.0004	
*Institution status*						
Single*	682 (42.9%)	102,520	74.1% (72.2–76.1)	–	< .0001	98.4%
Multi-institutional	907 (57.1%)	221,448	80.0% (78.5–81.5)	< .0001	0.515	
*Tier assessment*						
1*	824 (51.9%)	57,943	76.5% (74.8–78.2)	–	< .0001	98.4%
2	765 (48.1%)	266,025	78.7% (77.0–80.4)	0.076	0.0338	
*Masking*						
No masking*	341 (21.5%)	55,000	81.6% (79.1–83.9)	–	0.396	98.3%
Single	107 (6.73%)	22,081	71.8% (66.7–76.6)	0.0003	0.0003	
Double	389 (24.5%)	79,017	78.7% (76.3–81.0)	0.102	0.136	
Triple	287 (18.1%)	72,527	74.0% (71.0–76.9)	0.0001	< .0001	
Quadruple	465 (29.3%)	95,343	77.2% (75.0–79.4)	0.0103	0.0031	
*Study start year*						
2008*	121 (7.61%)	35,685	81.7% (77.5–85.5)	–	0.568	98.3%
2009	156 (9.82%)	30,239	81.1% (77.4–84.5)	0.828	0.750	
2010	134 (8.43%)	45,381	73.9% (69.5–78.1)	0.0098	0.0017	
2011	145 (9.13%)	17,765	72.5% (68.2–76.6)	0.0021	0.0001	
2012	159 (10.0%)	29,668	77.3% (73.4–81.0)	0.122	0.0910	
2013	189 (11.9%)	32,347	79.9% (76.5–83.2)	0.511	0.734	
2014	169 (10.6%)	25,349	75.6% (71.7–79.3)	0.0316	0.0082	
2015	177 (11.1%)	29,085	79.3% (75.7–82.7)	0.386	0.505	
2016	150 (9.44%)	41,432	80.4% (76.5–83.9)	0.637	0.942	
2017	128 (8.06%)	23,619	74.1% (69.6–78.4)	0.0136	0.0033	
2018	47 (2.96%)	10,243	72.7% (65.0–79.8)	0.0321	0.0304	
2019	14 (0.88%)	3155	79.4% (65.8–90.4)	0.731	0.864	

#### Multiracial

Relative to the Census (1.56%), multiracial participants were underrepresented (0.25%, 95% CI 0.21–0.31; *p* < 0.0001) in clinical trials overall, regardless of trial phase, institutional status, tier, and masking classification (Table 8). Of note, when examining masking status, multiracial representation did increase between trials with triple masking, relative to no masking (*p* = 0.025). Meanwhile, in multi-institutional trials multiracial representation significantly decreased, relative to single-institutional (*p* = 0.0001).

**Table 8:** Multiracial proportion estimates overall, by phase, multi-institutional status, tier assessment, masking, and year.

	Frequency (%), studies	Total number of participants	Estimated proportion (95% CI)	*P*-value compared to reference category (*)	*P*-value compared to 2010 Census proportion (1.56%)	${I}^{2}$(%)
*Multiracial*						
Overall	–	–	0.25% (0.21–0.31)	–	< 0.0001	83.9%
*Phase*						
II*	1094 (68.9%)	103,813	0.25% (0.19–0.32)	–	< .0001	83.8%
III	495 (31.2%)	220,155	0.26% (0.18–0.35)	0.894	< .0001	
*Institution status*						
Single*	682 (42.9%)	102,520	0.40% (0.30–0.51)	–	< .0001	83.5%
Multi-institutional	907 (57.1%)	221,448	0.19% (0.14–0.24)	0.0001	< .0001	
*Tier assessment*						
1*	824 (51.9%)	57,943	0.25% (0.18–0.33)	–	< .0001	83.9%
2	765 (48.1%)	266,025	0.26% (0.20–0.33)	0.855	< .0001	
*Masking*						
No masking*	341 (21.5%)	55,000	0.18% (0.10–0.29)	–	< .0001	83.5%
Single	107 (6.73%)	22,081	0.27% (0.11–0.51)	0.571	< .0001	
Double	389 (24.5%)	79,017	0.22% (0.14–0.32)	0.593	< .0001	
Triple	287 (18.1%)	72,527	0.37% (0.24–0.52)	0.0248	< .0001	
Quadruple	465 (29.3%)	95,343	0.27% (0.19–0.38)	0.176	< .0001	
*Study start year*						
2008*	121 (7.61%)	35,685	0.23% (0.09–0.42)	–	< .0001	83.1%
2009	156 (9.82%)	30,239	0.20% (0.08–0.35)	0.756	< .0001	
2010	134 (8.43%)	45,381	0.24% (0.11–0.44)	0.901	< .0001	
2011	145 (9.13%)	17,765	0.16% (0.05–0.31)	0.491	< .0001	
2012	159 (10.0%)	29,668	0.36% (0.20–0.57)	0.302	< .0001	
2013	189 (11.9%)	32,347	0.26% (0.13–0.43)	0.512	< .0001	
2014	169 (10.6%)	25,349	0.29% (0.15–0.48)	0.606	< .0001	
2015	177 (11.1%)	29,085	0.30% (0.16–0.49)	0.555	< .0001	
2016	150 (9.44%)	41,432	0.26% (0.13–0.44)	0.805	< .0001	
2017	128 (8.06%)	23,619	0.17% (0.05–0.34)	0.570	< .0001	
2018	47 (2.96%)	10,243	0.60% (0.25–1.11)	0.0772	< .0001	
2019	14 (0.88%)	3155	0.22% (0.00–1.04)	0.980	0.0053	

## Representation by disease strata: gender, ethnicity, and race

The 2977 clinical trials were also stratified by 44 disease categories and subcategories to examine variations in representation by gender, ethnicity, and race (Table 9; Supplemental Table 1).

**Table 9:**
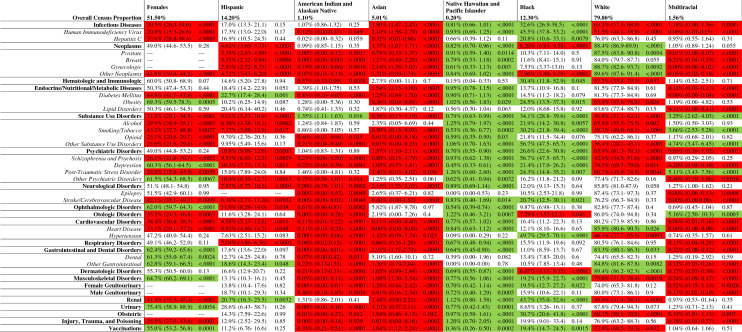
Representation of Gender, Ethnicity, and Race in Clinical Trials by Disease Categories and Sub-Categories.

Data presented in each cell includes the proportion of trial participants, 95% confidence interval, and corresponding P-value. A total of 44 disease categories/subcategories were analyzed. Relative to the US Census proportion, red cells represent underrepresentation of the demographic group, white cells appropriate representation, and green cells overrepresentation. Infectious diseases category excluded vaccination clinical trials; psychiatric disorders category excluded substance use disorders; cardiovascular disorders category excluded stroke/cerebrovascular disease; dermatologic disorders include cosmetic and plastic surgery clinical trials; female and male genitourinary categories excluded kidney and bladder diseases; urinary category excludes diseases of the kidney and genital organs.

Females were underrepresented in 18 disease strata and overrepresented in ten, relative to the Census proportion (51.5%, *p* < 0.05). Hispanics were underrepresented in 18 disease strata, while overrepresented in three (*p* < 0.05). Asians were underrepresented in 36 disease strata, appropriately represented in eight, and overrepresented in none. AIAN were underrepresented in 32 disease strata and overrepresented in 1 (*p* < 0.05). Multiracial participants were underrepresented in 25 disease strata (*p* < 0.05) and overrepresented in five. Whites were underrepresented in 17 disease strata and overrepresented in eight. Blacks were underrepresented in four disease strata and overrepresented in 20. NHPI were overrepresented in 37 disease strata (*p* < 0.05), and statistically underrepresented in none.

## Discussion

Over the last several decades, the issue of equitable clinical trial recruitment of women and minorities in the United States has garnered various degrees of attention^2–4,7^. In 1993, the NIH passed the Revitalization Act, which mandated inclusion of women and racial/ethnic minority groups in clinical trials^4,7^. The policy was then updated in 2000, 2001, and 2017 to require standardized minimum inclusion of sex, gender, and racial/ethnic minority groups in phase 3 clinical trials, with mandated reporting of demographic data to ClinicalTrials.gov^2–4^.

Despite FDA recommendations, our results indicate many studies did not comply with reporting guidance of demographic characteristics. Failure to report race and ethnicity data was prevalent in US clinical trials conducted between 2008 and 2019, a phenomenon reported in other reviews^1,21^. Likewise, the inclusion of sexual and gender minorities in clinical trials is nearly non-existent^25–28^. The lack of inclusion may be explained by incomplete and ambiguity in gender reporting on clinical trial recruitment servers, thus yielding in difficulties or failure to recruit from the population of sexual and gender minorities^29,30^.

Of the trials reporting demographics, these did not accurately represent the nation's demographics ^2–4^. When trials with significant gender skew were excluded from analysis, females remained underrepresented—a historically consistent observation^31^. The disparity likely arose secondary to a combination of research bias and categorization of women as a vulnerable population^32–35^. Nevertheless, there was a significant improvement in female representation from 2008 to 2018.

While female representation improved with time, such was not the case for underrepresented racial/ethnic groups, including Hispanics, AIAN, Asians, and multiracial populations. Low level of minority enrollment can potentially be explained by historical racial injustices, subject burden (i.e., transportation limitations, perceived interference with work/family obligations), lower socioeconomic status, communication barriers, and divergent cultural attitudes between investigators and participants^36–42^.

In contrast, Blacks and NHPI were found to be overrepresented overall in most clinical trials. These trends corroborate findings that people of color are much more willing to participate—as much as Whites–in trials than perceived^43–46^. Meanwhile, NHPI overrepresentation potentially represents an overall magnification of a small population, likely participating in trials from regions with significant NHPI density (i.e., Hawaii)^47^.

When examining clinical trial phase, females, Hispanics, and AIANs all exhibited greater representation in phase 3 than phase 2. Phase 3 trials may inherently lend themselves to readily attain diversity, as these investigations are typically more robust with larger financial resources and up to 1000 patients, relative to phase 2 trials which may have around a hundred participants^48,49^. On the other hand, the lower female proportion in phase 2 trials may arise secondary to phase 2 investigations often having exclusions on the basis of child-bearing potential^50^.

Hispanic, AIAN, Asian, and White groups have increased representation in tier 2 versus tier 1 trials, a trend possibly explained by increased trial size and multi-regionality. The difference in race/ethnicity based on trial size and multi-center nature, potentially highlights the trend of minorities to be differentially recruited based on trial characteristics—an issue raised in prior literature^1^.

When stratifying clinical trials by disease categories, our results suggested recruitment patterns often paralleled the baseline demographics of the particular illness. For instance, males were overrepresented in clinical trials investigating infectious diseases (i.e., HIV, hepatitis C), schizophrenia, cardiovascular diseases, stroke, and diabetes, while females were overrepresented in trials of musculoskeletal, gastrointestinal, obesity, and depression/mental health disorders^51–65^. Regarding race, representation paralleling the disease demographics was observed with overrepresentation of: Hispanics in diabetes and renal trials; AIAN in substance use disorders trials; Blacks in trials of infectious disease (i.e., HIV and hepatitis C), hypertension, stroke, obesity, hematology, musculoskeletal, and renal; Whites in gastrointestinal trials^66–75^.

## Limitations

**Figure 4:**
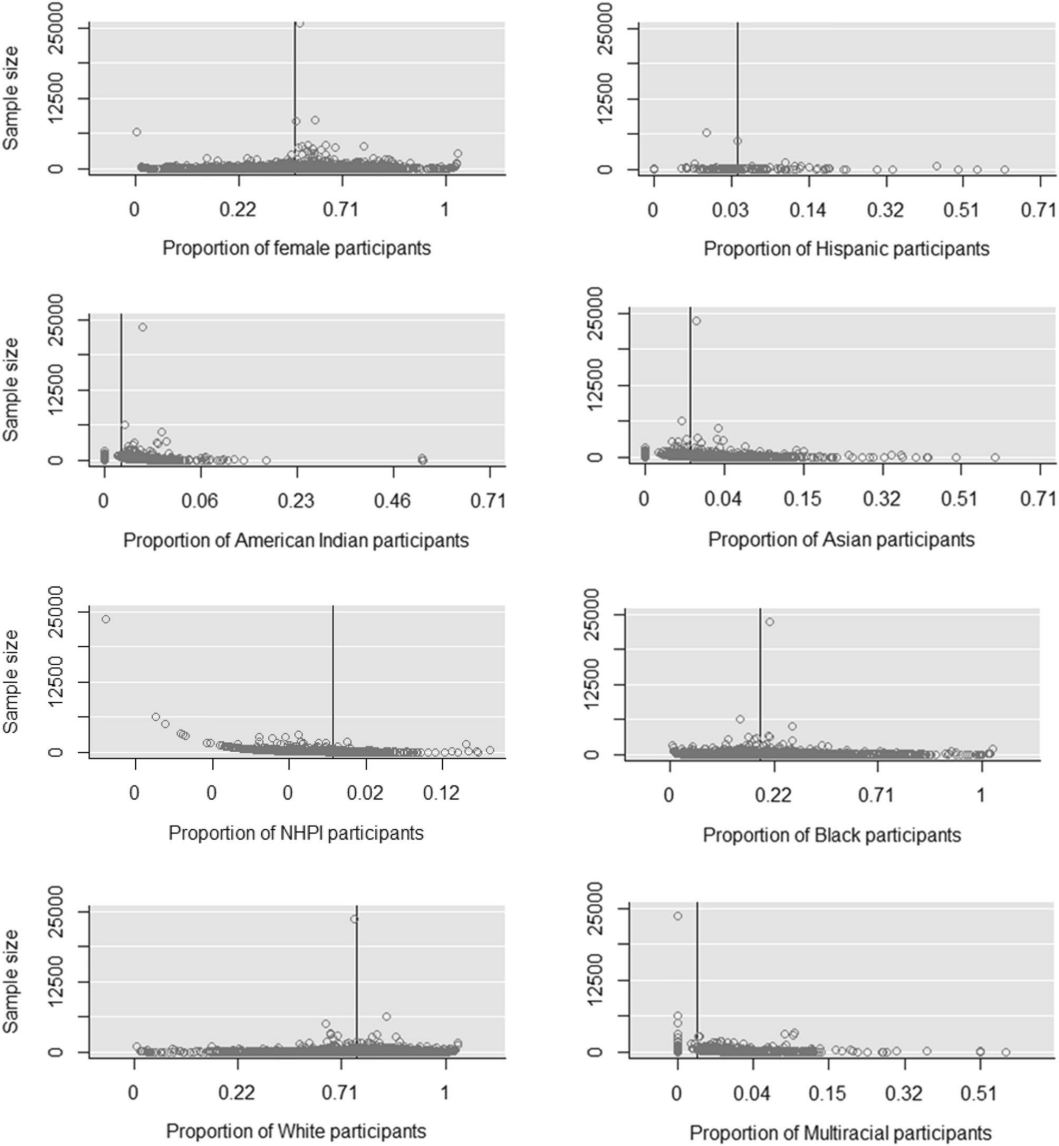
Funnel plots: Proportions of the subgroups (%) by sample size. The horizontal line is the summary proportion.

Overall, the findings should be considered in the context of several limitations. First, given non-compliance of data reporting on ClinicalTrials.gov, our investigation was unable appropriately conduct analyses stratified by age, while there is also a possibility studies omitted from the meta-analysis may have exhibited demographic proportions divergent from the observed trends. Second, reporting of race on ClinicalTrials.gov occasionally utilized non-standard categorization, requiring inference of race or exclusion of the data. Furthermore, given government policies to enhance reporting of race/ethnicity over the years for phase 3 clinical trials, some of the trends observed may have represented improved reporting rather than changes in demographic representation over the years. Finally, when examining funnel plots for gender, race, and ethnicity (Fig. 4), there appears a potential bias where the sample size of the study influences the proportion of multi-racial and NHPI proportions.

## Conclusion

The results of this study indicate persistence of gender, ethnic, and racial disparities in phase 2 and 3 randomized clinical trial recruitment of US adults. While representation of women and Hispanics has improved between 2008 to 2019, and Blacks with NHPIs generally overrepresented, the overall representation of several racial minorities (Asians, AIAN, and multi-racial individuals) has remained static, despite systems-based initiatives aimed at improving diversity. Overall, randomized clinical trials may not reflect the demographics of the populations sought to be served.

## Supplementary Information

Supplementary Information 1.

Supplementary Information 2.

Supplementary Information 3.

Supplementary Information 4.

## Abbreviations

AIAN: American Indian and Alaskan Native
CENTRAL: Cochrane Central Register of Controlled Trials
FDA: Food and Drug Administration
HIV: Human immunodeficiency virus
NIH: National Institutes of the Health
NHPI: Native Hawaiian and Pacific Islander
US: United States
WHO: World Health Organization
PRISMA: Preferred Reporting Items for Systematic Reviews and Meta-Analyses
