# Supplementary material for: Demographic recruitment bias of adults in United States randomized clinical trials by disease categories between 2008 to 2019: a systematic review and meta-analysis
Source: Sci Rep. 2023 Jan 2;13:42. doi: 10.1038/s41598-022-23664-1 (PMC9807581; doi:10.1038/s41598-022-23664-1)
Supplement: Supplementary file 1 — Supplementary Information 1. [file 41598_2022_23664_MOESM1_ESM.docx]

**Appendix**

**Pubmed (MEDLINE) Search Strategy**

1. United AND State*
2. United States
3. United AND States AND America
4. US* OR U.S.* OR USA OR U.S.A.
5. **1 OR 2 OR 3 OR 4**
6. clinical*
7. trial* OR trial OR trials
8. **6 AND 7**
9. random* OR randomized OR randomised
10. control* OR controlled OR control
11. **9 AND 10**
12. phase OR phases OR phase*
13. II* or II or ii or two
14. III* OR III OR iii OR three
15. **13 OR 14**
16. **12 AND 15**
17. **5 AND 8 AND 11 AND 16**

((((((United AND State*) OR (United States)) OR (United AND States AND America)) OR (US* OR U.S.* OR USA OR U.S.A.)) AND ((clinical*) AND (trial* OR trial OR trials))) AND ((random* OR randomized OR randomised) AND (control* OR controlled OR control))) AND ((phase OR phases OR phase*) AND (II* OR II OR ii OR two))

Search Date: October 14, 2020

**Embase Ovid Search Strategy**

1. ‘united states’
2. ‘clinical trial
3. ‘clinical study’
4. **2 OR 3**
5. ‘randomized controlled trial’
6. ‘phase 2 clinical trial’
7. ‘phase 3 clinical trial’
8. 6 OR 7
9. 1 AND 4 OR 5 OR 8

Search Date: October 14, 2020

**CENTRAL Search Strategy**

1. MeSH descriptor: [United States] explode all trees
2. United AND State*
3. United States
4. United AND States AND America
5. US* OR U.S.* OR USA OR U.S.A.
6. #1 OR #2 OR # 3 OR #4 OR #5
7. MeSH descriptor: [Clinical Trial] explode all trees
8. MeSH descriptor: [Clinical Study] explode all trees
9. clinical*
10. trial* OR trial OR trials
11. #9 AND #10
12. #7 OR #8 OR #11
13. MeSH descriptor: [Randomized Controlled Trial] explode all trees
14. random* OR randomized OR randomised
15. control* OR controlled OR control
16. #14 AND #15
17. #13 OR #16
18. phase OR phases OR phase*
19. II* or II or ii or two
20. III* OR III OR iii OR three
21. **19 OR 20**
22. **18 AND 21**

Search Date: October 14, 2020
