# Supplementary material for: Demographic recruitment bias of adults in United States randomized clinical trials by disease categories between 2008 to 2019: a systematic review and meta-analysis
Source: Sci Rep. 2023 Jan 2;13:42. doi: 10.1038/s41598-022-23664-1 (PMC9807581; doi:10.1038/s41598-022-23664-1)
Supplement: Supplementary file 4 — Supplementary Information 4. [file 41598_2022_23664_MOESM4_ESM.pdf]

Supplemental Table 1A. Infectious and Parasitic Diseases. Excluding vaccination clinical trials.

**Infectious and Parasitic Diseases**										
		**Overall (n=165)**			**HIV (n=76)**			**Hepatitis C (n=34)**		
Ethnicity Reported		45.5% (n=75)			50.0% (n=38)			50.% (n=17)		
Race Reported		63.0% (n=104)			68.4% (n=52)			61.8% (n=21)		
	Overall Census Proportion	Summary proportion (95% CI)	P-value	I^2 (%)	Summary proportion (95% CI)	P-value	I^2 (%)	Summary proportion (95% CI)	P-value	I^2 (%)
Females	51.5%	30.5% (26.4-34.6)	<.0001	97.4%	20.8% (15.3-26.8)	<.0001	97.2%	32.6% (28.8-36.4)	<.0001	83.5%
Hispanic	14.2%	17.0% (13.3- 21.1)	0.15	96.9%	17.3% (13.0- 22.0)	0.17	92.5%	16.9% (10.5- 24.5)	0.44	96.0%
AIAN	1.10%	1.07% (0.86-1.32)	0.25	12.3%	0.12% (0.00-0.37)	0.049	26.4%	0.02% (0.00- 0.32)	0.059	32.0%
Asian	5.01%	1.90% (1.47- 2.45)	<.0001	64.8%	2.10% (1.58- 2.79)	0.0008	32.1%	0.32% (0.01-0.90)	<.0001	42.6%
NHPI	0.20%	0.81% (0.66- 1.01)	<.0001	0.00%	0.93% (0.69- 1.25)	<.0001	0%	0.66% (0.39- 1.12)	0.11	0%
Black	12.3%	32.6% (26.9-38.5)	<.0001	98.2%	45.5% (37.8- 53.2)	<.0001	96.8%	20.8% (10.6- 33.1)	0.0079	98.0%
White	79.8%	63.2% (57.3- 68.9)	<.0001	98.0%	51.5% (44.1- 58.9)	<.0001	96.5%	76.0% (63.3- 86.8)	0.45	98.0%
Multiracial	1.56%	1.18% (0.90- 1.56)	0.0081	52.6%	0.06% (0.01-0.15)	<.0001	9.82%	0.95% (0.55- 1.64)	0.31	3.10%

Supplemental Table 1B. Neoplasms and Chemotherapy.

Neoplasms and Chemotherapy																
		**Overall (n=406)**			**Prostate cancer (n=54)**			**Breast cancer (n=60)**			**Gynecologic cancer (n=21)**			**Other neoplasms (n=271)**		
Ethnicity Reported		38.7% (n=157)			42.6% (n=23)			38.3% (n=23)			42.9% (n=9)			37.6% (n=102)		
Race Reported		64.0% (n=260)			72.2% (n=39)			65.0% (n=39)			76.2% (n=16)			57.6% (n=156)		
	Overall Census Proportion	Summary proportion (95% CI)	P-value	I^2 (%)	Summary proportion (95% CI)	P-value	I^2 (%)	Summary proportion (95% CI)	P-value	I^2 (%)	Summary proportion (95% CI)	P-value	I^2 (%)	Summary proportion (95% CI)	P-value	I^2 (%)
Females	51.5%	49.0% (44.6- 53.5)	0.28	99.1%	---	---	---	---	---	---	---	---	---	41.8% (39.4- 44.2)	<.0001	94.6%
Hispanic	14.2%	4.62% (3.60- 5.75)	<.0001	93.4%	3.35% (2.03- 4.99)	<.0001	59.0%	5.35% (2.12- 9.94)	0.0006	95.3%	5.33% (2.72- 8.75)	<.0001	89.7%	4.72% (3.43- 6.20)	<.0001	92.8%
AIAN	1.10%	0.99% (0.85- 1.15)	0.35	14.3%	0.00% (0.00- 0.12)	0.0017	0%	0.00% (0.00- 0.00)	<.0001	6.33%	0.19% (0.00- 0.66)	0.0016	71.1%	0.07% (0.03- 0.13)	<.0001	28.7%
Asian	5.01%	1.37% (1.07- 1.71)	<.0001	82.7%	0.78% (0.33- 1.37)	<.0001	24.9%	1.27% (0.49- 2.29)	<.0001	76.7%	2.14% (1.38- 3.02)	<.0001	29.5%	1.31% (0.89-1.79)	<.0001	77.1%
NHPI	0.20%	0.82% (0.70- 0.96)	<.0001	0.00%	0.91% (0.59- 1.40)	0.0114	0%	0.79% (0.53- 1.18)	0.0002	0%	0.61% (0.33- 1.13)	0.0030	0%	0.84% (0.69- 1.02)	<.0001	0.00%
Black	12.3%	8.20% (6.91- 9.58)	<.0001	94.4%	10.3% (7.11- 14.0)	0.50	87.4%	11.6% (8.41- 15.1)	0.91	91.5%	7.53% (3.37-13.0)	0.13	95.3%	7.06% (5.49- 8.79)	<.0001	94.5%
White	79.8%	88.4% (86.9-89.9)	<.0001	94.3%	87.5% (83.8- 90.8)	0.0004	86.1%	84.0% (79.7- 87.7)	0.055	93.0%	88.7% (82.6- 93.7)	0.0092	95.1%	89.6% (87.6- 91.4)	<.0001	94.5%
Multiracial	1.56%	1.05% (0.89- 1.24)	0.055	19.0%	0.01% (0.00- 0.07)	<.0001	0%	0.21% (0.04- 0.53)	<.0001	67.2%	0.00% (0.00- 0.02)	<.0001	0%	0.05% (0.02- 0.10)	<.0001	27.05%

Supplemental Table 1C. Diseases of the blood/blood forming organs and disorders involving the immune mechanisms.

**Blood and Immunologic Disorders (n=35)**				
Ethnicity Reported	28.6% (n=10)			
Race Reported	42.9% (n=15)			
	Overall Census Proportion[1]	Summary Proportion (95% CI)	P-value	I^2 (%)
Females	51.5%	60.0% (50.8- 68.9)	0.070	98.5%
Hispanic	14.2%	14.6% (5.20- 27.8)	0.94	97.6%
AIAN	1.10%	0.57% (0.33-0.99)	0.0038	0%
Asian	5.01%	2.73% (0.00- 11.1)	0.70	97.9%
NHPI	0.20%	0.15% (0.04- 0.33)	0.53	0%
Black	12.3%	30.4% (11.8- 52.9)	0.045	99.0%
White	79.8%	57.3% (33.8- 79.3)	0.037	99.1%
Multiracial	1.56%	1.14% (0.52- 2.51)	0.71	51.3%

Supplemental Table 1D. Endocrine, nutritional, and metabolic diseases.

**Endocrine, Nutritional, and Metabolic Diseases**													
		**Overall (n=268)**			**Diabetes and Insulin Resistance (n=175)**			**Obesity (n=40)**			**Dyslipidemias (n=27)**		
Ethnicity Reported		35.8% (n=96)			38.9% (n=68)			27.5% (n=11)			29.6% (n=8)		
Race Reported		57.8% (n=155)			61.7% (n=108)			50.0% (n=20)			48.1% (n=13)		
	Overall Census Proportion	Summary Proportion (95% CI)	P-value	I^2 (%)	Summary proportion (95% CI)	P-value	I^2 (%)	Summary proportion (95% CI)	P-value	I^2 (%)	Summary proportion (95% CI)	P-value	I^2 (%)
Females	51.5%	50.3% (47.4- 53.3)	0.44	96.6%	44.8% (41.7- 47.8)	<.0001	94.6%	69.3% (59.5-78.3)	0.0005	98.3%	50.3% (46.1- 54.5)	0.59	90.6%
Hispanic	14.2%	18.4% (14.2- 22.9)	0.051	97.3%	22.7% (17.4- 28.4)	0.0010	97.5%	10.2% (6.25- 14.9)	0.087	73.6%	20.4% (6.14- 40.2)	0.46	97.9%
AIAN	1.10%	1.39% (1.10-1.75)	0.53	57.4%	0.43% (0.26- 0.65)	<.0001	61.1%	1.28% (0.00- 5.36)	0.3011	93.7%	0.74% (0.41- 1.33)	0.32	0%
Asian	5.01%	2.54% (2.15- 3.00)	<.0001	54.7%	1.25% (0.84- 1.70)	<.0001	65.2%	0.36% (0.04- 0.88)	<.0001	0%	1.87% (0.30- 4.37)	0.12	88.9%
NHPI	0.20%	0.95% (0.78- 1.15)	<.0001	6.09%	0.90% (0.71- 1.13)	<.0001	10.1%	1.02% (0.56- 1.83)	0.029	0%	0.56% (0.30- 1.04)	0.063	0%
Black	12.3%	13.7% (10.9- 16.8)	0.10	96.7%	14.5% (11.2- 18.2)	0.079	97.0%	24.5% (13.5- 37.3)	0.015	96.6%	12.0% (8.68- 15.8)	0.92	81.8%
White	79.8%	81.5% (77.9- 84.9)	0.61	97.2%	81.3% (77.3- 84.9)	0.69	96.9%	63.9% (47.6- 78.9)	0.026	97.6%	83.6% (77.4- 88.7)	0.15	92.0%
Multiracial	1.56%	0.12% (0.03- 0.27)	<.0001	80.3%	0.00% (0.00- 0.04)	<.0001	52.0%	1.19% (0.00- 4.82)	0.53	92.5%	0.11% (0.00- 0.41)	<.0001	39.6%

Supplemental Table 1E. Substance use disorders.

**Substance Use Disorders**																
		**Overall (n=245)**			**Alcohol Dependence (n=58)**			**Smoking, Tobacco, and Nicotine Dependence (n=75)**			**Opioid Dependence (n=38)**			**Other Substances (n=74)**		
Ethnicity Reported		100% (n=88)			100% (n=18)			100% (n=31)			100% (n=16)			100% (n=23)		
Race Reported		100% (n=137)			100% (n=32)			100% (n=43)			100% (n=22)			100% (n=40)		
	Overall Census Proportion	Summary Proportion (95% CI)	P-value	I^2 (%)	Summary proportion (95% CI)	P-value	I^2 (%)	Summary proportion (95% CI)	P-value	I^2 (%)	Summary proportion (95% CI)	P-value	I^2 (%)	Summary proportion (95% CI)	P-value	I^2 (%)
Females	51.5%	31.8% (29.1- 34.5)	<.0001	96.9%	29.9% (24.9- 35.1)	<.0001	93.1%	43.2% (37.7- 48.8)	0.0037	98.5%	25.1% (20.6- 29.7)	<.0001	90.9%	25.6% (21.6- 29.8)	<.0001	93.5%
Hispanic	14.2%	8.01% (5.53- 10.9)	<.0001	98.1%	6.30% (3.38- 10.1)	0.0002	80.8%	7.17% (3.08- 12.8)	0.017	99.2%	9.70% (2.76- 20.3)	0.36	98.0%	9.95% (5.49- 15.6)	0.13	95.6%
AIAN	1.10%	1.35% (1.11- 1.63)	0.016	43.7%	1.24% (0.84- 1.83)	0.59	0%	0.86% (0.00- 3.05)	0.57	98.6%	0.40% (0.07- 0.99)	0.025	74.5%	0.21% (0.04- 0.49)	<.0001	51.1%
Asian	5.01%	0.58% (0.15- 1.19)	<.0001	93.0%	2.35% (0.05- 6.69)	0.44	95.2%	0.50% (0.15- 0.98)	<.0001	79.5%	0.01% (0.00- 0.18)	<.0001	20.0%	0.01% (0.00- 0.23)	<.0001	36.3%
NHPI	0.20%	0.79% (0.63- 0.99)	<.0001	10.4%	1.25% (0.79- 1.97)	<.0001	3.52%	0.53% (0.36- 0.77)	0.0002	4.92%	0.59% (0.35- 0.99)	0.030	0%	1.06% (0.70- 1.63)	<.0001	13.8%
Black	12.3%	34.1% (28.8- 39.6)	<.0001	98.8%	21.9% (14.2- 30.8)	0.0057	95.5%	30.2% (21.8- 39.4)	<.0001	99.2%	21.8% (11.5- 34.4)	0.076	98.6%	56.7% (47.5- 65.7)	<.0001	97.0%
White	79.8%	56.8% (51.1- 62.4)	<.0001	98.8%	65.8% (55.5- 75.5)	0.0020	96.0%	58.7% (49.0- 68.1)	<.0001	99.2%	75.1% (62.2- 86.1)	0.37	98.5%	36.4% (28.1- 45.1)	<.0001	96.7%
Multiracial	1.56%	3.25% (2.62- 4.03)	<.0001	89.2%	1.50% (0.50- 3.03)	0.93	83.0%	3.66% (2.53- 5.28)	<.0001	95.1%	1.17% (0.68- 2.01)	0.82	46.1%	4.74% (3.47- 6.45)	<.0001	67.4%

Supplemental Table 1F. Mental, behavioral, and neurodevelopmental disorders. Excluding substance use disorders.

**Psychiatric Disorders**																
		**Overall (n=215)**			**Schizophrenia and Psychosis (n=44)**			**Depression (n=79)**			**PTSD (n=30)**			**Other Mental Disorders (n=62)**		
Ethnicity Reported		40.9% (n=88)			45.5% (n=20)			34.2% (n=27)			43.3% (n=13)			100% (n=28)		
Race Reported		57.2% (n=123)			70.5% (n=31)			49.4% (n=39)			60.0% (n=18)			100% (n=35)		
	Overall Census Proportion	Summary proportion (95% CI)	P-value	I^2 (%)	Summary proportion (95% CI)	P-value	I^2 (%)	Summary proportion (95% CI)	P-value	I^2 (%)	Summary proportion (95% CI)	P-value	I^2 (%)	Summary proportion (95% CI)	P-value	I^2 (%)
Females	51.5%	49.0% (44.8- 53.2)	0.24	98.4%	26.1% (21.8- 30.7)	<.0001	91.5%	60.3% (56.1-64.5)	<.0001	96.7%	28.9% (15.8- 43.9)	0.0035	98.6%	61.5% (54.3- 68.5)	0.0067	98.3%
Hispanic	14.2%	10.8% (9.08- 12.6)	0.0003	92.6%	9.53% (6.91- 12.5)	0.0029	75.1%	10.3% (7.71- 13.3)	0.011	94.7%	15.0% (7.89- 24.0)	0.84	93.3%	10.4% (8.35- 12.7)	0.0013	82.6%
AIAN	1.10%	1.04% (0.83- 1.31)	0.89	59.0%	0.23% (0.06- 0.50)	<.0001	35.6%	0.22% (0.09- 0.39)	0.0041	20.9%	1.46% (0.00- 4.81)	0.32	92.6%	0.75% (0.56- 1.01)	0.014	0%
Asian	5.01%	1.57% (1.10- 2.11)	<.0001	88.7%	0.80% (0.15- 1.79)	<.0001	70.4%	1.05% (0.73- 1.41)	<.0001	45.2%	1.40% (0.03- 4.02)	0.036	88.9%	1.25% (0.35- 2.51)	0.061	86.6%
NHPI	0.20%	0.70% (0.55- 0.90)	<.0001	34.0%	0.93% (0.62- 1.38)	<.0001	0%	0.45% (0.33- 0.61)	<.0001	0%	1.26% (0.60- 2.60)	<.0001	55.1%	0.62% (0.41- 0.94)	0.0032	10.0%
Black	12.3%	26.6% (22.6- 30.8)	<.0001	97.9%	56.7% (47.5- 65.7)	<.0001	95.8%	21.4% (17.0- 26.2)	<.0001	97.0%	24.3% (14.8- 35.2)	0.0070	95.8%	16.2% (11.8- 21.2)	0.090	96.4%
White	79.8%	65.9% (61.3- 70.3)	<.0001	98.1%	42.9% (34.5- 51.6)	<.0001	95.8%	74.5% (69.7- 79.0)	0.011	96.7%	60.7% (45.9- 74.5)	0.0041	97.3%	77.4% (71.7- 82.6)	0.16	96.0%
Multiracial	1.56%	0.65% (0.36- 1.02)	<.0001	88.8%	0.97% (0.29- 2.05)	0.25	84.1%	0.20% (0.04- 0.48)	<.0001	83.8%	5.11% (3.43- 7.56)	<.0001	83.8%	0.46% (0.10- 1.06)	0.0016	86.8%

Supplemental Table 1G. Diseases of the nervous system. Including stroke/cerebrovascular disease, and pain disorders.

**Neurological Disorders**										
		**Overall (n=256)**			**Epilepsy (n=7)**			**Stroke and Cerebrovascular Disease (n=31)**		
Ethnicity Reported		34.4% (n=88)			14.3% (n=1)			25.8% (n=8)		
Race Reported		51.6% (n=132)			71.4% (n=5)			58.1% (n=18)		
	Overall Census Proportion	Summary Proportion (95% CI)	P-value	I^2 (%)	Summary proportion (95% CI)	P-value	I^2 (%)	Summary proportion (95% CI)	P-value	I^2 (%)
Females	51.5%	51.% (48.1- 54.8)	0.95	98.0%	51.5% (42.9- 60.1)	0.99	39.8%	42.1% (35.1- 49.3)	0.0099	96.0%
Hispanic	14.2%	7.97% (5.77- 10.5)	<.0001	98.1%	---	---	---	6.30% (2.71- 11.26)	0.0032	96.3%
AIAN	1.10%	0.90% (0.79- 1.01)	0.0002	0.55%	0.00% (0.00- 0.53)	0.0049	0%	0.00% (0.00- 0.02)	0.0049	39.0%
Asian	5.01%	2.15% (1.75- 2.65)	<.0001	72.3%	2.65% (0.37- 6.21)	0.82	0%	0.60% (0.00-1.82)	<.0001	83.3%
NHPI	0.20%	0.89% (0.69-1.14)	<.0001	49.6%	0.00% (0.00-0.53)	0.23	0%	0.83% (0.40- 1.69)	0.014	42.1%
Black	12.3%	12.0% (9.13- 15.3)	0.64	98.4%	10.5% (2.53-21.8)	0.99	69.6%	20.7% (12.5- 30.1)	0.021	98.1%
White	79.8%	85.8% (81.0-87.9)	0.058	98.4%	87.4% (73.1- 97.3)	0.37	78.1%	76.2% (66.3- 84.9)	0.33	98.1%
Multiracial	1.56%	1.27% (1.00- 1.62)	0.21	55.1%	0.00% (0.00- 0.53)	0.0008	0%	0.00% (0.00 0.00)	<.0001	28.0%

Supplemental Table 1H. Diseases of the eye and adnexa.

**Ophthalmologic Disorders (n=158)**				
Ethnicity Reported	26.6% (n=42)			
Race Reported	40.5% (n=64)			
	Overall Census Proportion	Summary Proportion (95% CI)	P-value	I^2 (%)
Females	51.5%	62.0% (59.7- 64.3)	<.0001	94.7%
Hispanic	14.2%	11.0% (8.29- 14.0)	0.038	95.6%
AIAN	1.10%	0.61% (0.46-0.83)	0.0062	24.9%
Asian	5.01%	5.82% (1.87- 9.78)	0.97	99.9%
NHPI	0.20%	0.54% (0.39-0.74)	<.0001	19.3%
Black	12.3%	9.87% (6.98- 13.1)	0.38	97.3%
White	79.8%	82.8% (77.7- 87.4)	0.40	98.4%
Multiracial	1.56%	0.69% (0.45- 1.04)	0.87	71.7%

Supplemental Table 1I. Diseases of the ear and mastoid process.

**Otologic Diseases (n=11)**				
Ethnicity Reported	45.5% (n=5)			
Race Reported	45.5% (n=5)			
	Overall Census Proportion	Summary Proportion (95% CI)	P-value	I^2 (%)
Females	51.5%	35.2% (24.3- 46.8)	0.0063	93.1%
Hispanic	14.2%	11.6% (3.28- 24.1)	0.64	88.9%
AIAN	1.10%	0.00% (0.00- 0.28)	<.0001	0%
Asian	5.01%	2.19% (0.00- 7.26)	0.40	74.4%
NHPI	0.20%	1.22% (0.46- 3.21)	0.0067	0%
Black	12.3%	7.29% (3.57-12.2)	0.045	53.7%
White	79.8%	86.0% (74.0- 94.8)	0.34	85.2%
Multiracial	1.56%	5.16% (2.50- 10.3)	0.0003	34.9%

Supplemental Table 1J. Diseases of the circulatory system. Excluded stroke and cerebrovascular diseases.

**Cardiovascular Disorders**										
		**Overall (n=144)**			**Heart Disease (n=131)**			**Hypertension (n=13)**		
Ethnicity Reported		22.9% (n=33)			19.8% (n=26)			53.8% (n=7)		
Race Reported		50.0% (n=72)			46.6% (n=61)			84.6% (n=11)		
	Overall Census Proportion	Summary Proportion (95% CI)	P-value	I^2 (%)	Summary proportion (95% CI)	P-value	I^2 (%)	Summary proportion (95% CI)	P-value	I^2 (%)
Females	51.5%	34.4% (30.6- 38.3)	<.0001	96.8%	33.1% (29.1- 37.2)	<.0001	96.4%	47.2% (40.0- 54.4)	0.24	94.1%
Hispanic	14.2%	8.58% (5.13- 12.8)	0.011	94.8%	8.92% (4.80- 14.2)	0.048	95.2%	7.63% (2.51- 15.2)	0.083	89.0%
AIAN	1.10%	0.11% (0.04- 0.22)	<.0001	38.8%	0.11% (0.03- 0.23)	<.0001	39.9%	0.00% (0.00- 0.06)	<.0001	31.9%
Asian	5.01%	0.11% (0.00- 0.40)	<.0001	66.1%	0.00% (0.00- 0.08)	<.0001	34.7%	1.42% (0.09- 3.74)	0.023	90.9%
NHPI	0.20%	0.77% (0.57- 1.02)	<.0001	9.31%	0.84% (0.63- 1.12)	<.0001	0%	0.09% (0.00- 0.29)	0.22	33.5%
Black	12.3%	16.4% (11.2- 22.3)	0.13	98.6%	12.1% (8.10- 16.6)	0.65	96.7%	49.7% (29.5- 70.1)	<.0001	99.2%
White	79.8%	80.2% (73.9- 85.9)	0.86	98.4%	85.9% (80.6- 90.5)	0.026	97.6%	46.3% (27.7- 65.5)	0.0003	99.1%
Multiracial	1.56%	0.06% (0.01- 0.16)	<.0001	56.9%	0.00% (0.00- 0.00)	<.0001	45.5%	0.74% (0.35- 1.57)	0.61	26.6%

Supplemental Table 1K. Respiratory system disorders.

**Diseases of the respiratory system (n=152)**				
Ethnicity Reported	33.6% (n=51)			
Race Reported	50.0% (n=76)			
	Overall Census Proportion	Summary Proportion (95% CI)	P-value	I^2 (%)
Females	51.5%	49.1% (46.2- 52.0)	0.11	96.1%
Hispanic	14.2%	5.04% (3.86- 6.36)	<.0001	89.1%
AIAN	1.10%	0.06% (0.02-0.12)	<.0001	28.8%
Asian	5.01%	0.66% (0.26-1.20)	<.0001	83.8%
NHPI	0.20%	0.67% (0.48- 0.94)	<.0001	29.5%
Black	12.3%	15.5% (11.9- 19.6)	0.092	97.7%
White	79.8%	80.5% (76.1- 84.6)	0.95	97.6%
Multiracial	1.56%	0.13% (0.04- 0.28)	<.0001	69.2%

Supplemental Table 1L. Diseases of the digestive system

**Gastrointestinal and Dental Disorders**										
		**Overall (n=208)**			**Dental Diseases (n=59)**			**Other Gastrointestinal Diseases (n=149)**		
Ethnicity Reported		30.8% (n=64)			16.9% (n=10)			36.2% (n=54)		
Race Reported		39.9% (n=83)			25.4% (n=15)			45.6% (n=68)		
	Overall Census Proportion	Summary Proportion (95% CI)	P-value	I^2 (%)	Summary proportion (95% CI)	P-value	I^2 (%)	Summary proportion (95% CI)	P-value	I^2 (%)
Females	51.5%	62.4% (59.2- 65.6)	<.0001	97.7%	61.3% (55.0- 67.4)	0.0024	93.4%	62.8% (59.1- 66.5)	<.0001	98.0%
Hispanic	14.2%	17.6% (13.6- 22.0)	0.097	97.8%	12.7% (4.25- 24.8)	0.78	96.7%	18.6% (14.3- 23.4)	0.048	97.8%
AIAN	1.10%	0.00% (0.00- 0.01)	<.0001	5.35%	0.07% (0.00-0.42)	0.031	0%	0.22% (0.13-0.33)	<.0001	34.9%
Asian	5.01%	1.91% (1.17-2.79)	<.0001	89.8%	5.10% (1.60- 10.1)	0.72	91.4%	1.36% (0.79-2.04)	<.0001	84.8%
NHPI	0.20%	0.64% (0.45-0.90)	<.0001	48.4%	0.39% (0.00- 1.96)	0.082	81.4%	0.00% (0.00-0.00)	0.78	9.78%
Black	12.3%	11.0% (8.59- 13.7)	0.67	96.0%	13.4% (7.83- 20.0)	0.60	90.6%	10.5% (7.85- 13.4)	0.48	95.5%
White	79.8%	83.3% (80.1- 86.3)	0.033	96.6%	74.4% (65.5- 82.3)	0.15	92.1%	84.8% (81.6- 87.8)	0.0082	96.2%
Multiracial	1.56%	0.22% (0.08- 0.42)	<.0001	79.4%	1.25% (0.19- 2.92)	0.59	59.1%	0.12% (0.03- 0.26)	<.0001	72.2%

Supplemental Table 1M. Diseases of the skin/subcutaneous tissue. Including cosmetic and plastic surgery clinical trials.

**Dermatologic Disorders (n=170)**				
Ethnicity Reported	41.1% (n=70)			
Race Reported	53.5% (n=91)			
	Overall Census Proportion	Summary Proportion (95% CI)	P-value	I^2 (%)
Females	51.5%	55.3% (50.5- 60.0)	0.13	98.7%
Hispanic	14.2%	16.6% (12.9-20.7)	0.22	97.9%
AIAN	1.10%	0.21% (0.12-0.33)	<.0001	55.0%
Asian	5.01%	1.65% (0.99- 2.44)	<.0001	90.6%
NHPI	0.20%	0.69% (0.55- 0.87)	<.0001	7.27%
Black	12.3%	6.07% (4.11- 8.35)	<.0001	97.0%
White	79.8%	89.4% (86.2- 92.3)	<.0001	97.7%
Multiracial	1.56%	0.17% (0.07- 0.30)	<.0001	68.5%

Supplemental Table 1N. Diseases of the musculoskeletal system and connective tissues.

**Musculoskeletal Disorders (n=167)**				
Ethnicity Reported	32.3% (n=54)			
Race Reported	44.9% (n=75)			
	Overall Census Proportion	Summary Proportion (95% CI)	P-value	I^2 (%)
Females	51.5%	64.7% (60.2- 69.1)	<.0001	98.8%
Hispanic	14.2%	13.1% (10.3- 16.1)	0.45	95.9%
AIAN	1.10%	0.03% (0.00- 0.11)	<.0001	40.9%
Asian	5.01%	1.88% (1.30- 2.54)	<.0001	81.5%
NHPI	0.20%	0.77% (0.56- 1.06)	<.0001	52.1%
Black	12.3%	19.2% (15.9- 22.7)	<.0001	96.4%
White	79.8%	75.0% (71.5- 78.4)	0.0019	95.8%
Multiracial	1.56%	0.24% (0.09- 0.47)	<.0001	80.9%

Supplemental Table 1O. Female genitourinary disease. Excluding diseases of the kidney and bladder.

**Female genitourinary disease (n=73)**				
Ethnicity Reported	100% (n=33)			
Race Reported	100% (n=45)			
	Overall Census Proportion	Summary Proportion (95% CI)	P-value	I^2 (%)
Hispanic	14.2%	13.8% (10.4- 17.6)	0.82	95.3%
AIAN	1.10%	0.00% (0.00- 0.01)	<.0001	22.8%
Asian	5.01%	1.28% (0.44- 2.42)	<.0001	91.3%
NHPI	0.20%	0.70% (0.42- 1.14)	<.0001	64.2%
Black	12.3%	19.5% (12.7- 27.2)	0.022	98.7%
White	79.8%	74.0% (65.3- 81.8)	0.12	98.8%
Multiracial	1.56%	0.52% (0.15- 1.13)	0.0026	90.0%

Supplemental Table 1P. Male genitourinary disease. Excluding diseases of the kidney and bladder.

**Male non-neoplastic genitourinary disease (n=36)**				
Ethnicity Reported	100% (n=10)			
Race Reported	100% (n=16)			
	Overall Census Proportion	Summary Proportion (95% CI)	P-value	I^2 (%)
Hispanic	14.2%	18.7% (10.1- 29.3)	0.34	96.1%
AIAN	1.10%	0.46% (0.19- 0.83)	0.0019	22.5%
Asian	5.01%	0.91% (0.06- 2.34)	0.0001	80.0%
NHPI	0.20%	0.72% (0.40- 1.29)	0.0005	0.00%
Black	12.3%	15.9% (10.6- 22.1)	0.11	92.6%
White	79.8%	80.0% (73.1- 86.1)	0.90	93.2%
Multiracial	1.56%	0.17% (0.02- 0.49)	<.0001	44.7%

Supplemental Table 1Q. Renal diseases.

**Renal disease (n=75)**				
Ethnicity Reported	45.3% (n=34)			
Race Reported	52.0% (n=39)			
	Overall Census Proportion	Summary Proportion (95% CI)	P-value	I^2 (%)
Females	51.5%	41.4% (37.5- 45.4)	<.0001	95.0%
Hispanic	14.2%	20.7% (16.3- 25.5)	0.0032	94.9%
AIAN	1.10%	1.31% (0.86- 2.01)	0.41	62.0%
Asian	5.01%	1.44% (0.80-2.21)	<.0001	65.8%
NHPI	0.20%	1.12% (0.80- 1.58)	<.0001	22.4%
Black	12.3%	43.7% (35.0- 52.6)	<.0001	98.0%
White	79.8%	49.9% (41.4- 58.4)	<.0001	97.8%
Multiracial	1.56%	0.93% (0.53- 01.64)	0.35	61.8%

Supplemental Table 1R. Urinary Diseases. Excluding diseases of the kidney and genital organs.

**Urinary Diseases (n=20)**				
Ethnicity Reported	25.0% (n=5)			
Race Reported	70.0% (n=14)			
	Overall Census Proportion	Summary Proportion (95% CI)	P-value	I^2 (%)
Females	51.5%	75.4% (58.9- 88.9)	0.0054	98.9%
Hispanic	14.2%	28.6% (6.43- 58.7)	0.26	98.1%
AIAN	1.10%	0.00% (0.00- 0.16)	<.0001	0.32%
Asian	5.01%	1.10% (0.37-2.11)	<.0001	50.0%
NHPI	0.20%	0.77% (0.42-1.43)	0.0001	0%
Black	12.3%	8.65% (3.26- 16.1)	0.3661	95.3%
White	79.8%	87.8% (79.4- 94.3)	0.073	95.4%
Multiracial	1.56%	1.23% (0.71- 2.13)	0.41	12.6%

Supplemental Table 1S. Pregnancy, childbirth, and puerperium.

**Obstetrics (n=40)**				
Ethnicity Reported	42.5% (n=17)			
Race Reported	55.0% (n=22)			
	Overall Census Proportion	Summary Proportion (95% CI)	P-value	I^2 (%)
Hispanic	14.2%	14.3% (7.59- 22.6)	0.99	95.1%
AIAN	1.10%	0.01% (0.00- 0.27)	0.012	21.1%
Asian	5.01%	1.94% (0.46- 4.13)	0.042	85.6%
NHPI	0.20%	0.97% (0.58- 1.61)	<.0001	1.54%
Black	12.3%	30.7% (20.6- 41.8)	<.0001	96.5%
White	79.8%	60.7% (50.1- 70.9)	<.0001	95.9%
Multiracial	1.56%	0.33% (0.01- 1.13)	0.0070	79.6%

Supplemental Table 1T. Injury, trauma and poisoning.

**Injury, Trauma, and Poisoning (n=56)**				
Ethnicity Reported	23.2% (n=13)			
Race Reported	39.3% (n=22)			
	Overall Census Proportion	Summary Proportion (95% CI)	P-value	I^2 (%)
Females	51.5%	35.5% (27.8- 43.6)	0.0001	98.1%
Hispanic	14.2%	12.9% (2.52- 29.5)	0.85	98.5%
AIAN	1.10%	0.00% (0.00- 0.14)	0.029	46.9%
Asian	5.01%	0.01% (0.00- 0.16)	<.0001	5.05%
NHPI	0.20%	1.20% (0.70- 2.05)	<.0001	34.2%
Black	12.3%	19.9% (9.01- 33.4)	0.14	98.6%
White	79.8%	76.9% (63.2- 88.3)	0.56	98.5%
Multiracial	1.56%	0.18% (0.01- 0.57)	<.0001	62.4%

Supplemental Table 1U. Vaccinations.

**Vaccinations (n=77)**				
Ethnicity Reported	37.7% (n=29)			
Race Reported	50.6% (n=39)			
	Overall Census Proportion	Summary Proportion (95% CI)	P-value	I^2 (%)
Females	51.5%	55.0% (53.2- 56.8)	0.0001	95.5%
Hispanic	14.2%	11.2% (6.76- 16.6)	0.25	99.1%
American Indian	1.10%	0.35% (0.21- 0.51)	<.0001	50.7%
Asian	5.01%	1.64% (1.12- 2.24)	<.0001	89.1%
NHPI	0.20%	0.36% (0.26- 0.50)	0.0002	35.1%
Black	12.3%	19.4% (14.7- 24.5)	0.0015	98.9%
White	79.8%	73.9% (68.5- 79.3)	0.032	99.3%
Multiracial	1.56%	1.04% (0.64- 1.66)	0.51	91.8%
